# Intracellular protein glycosylation modulates insulin mediated lifespan in C. elegans

**DOI:** 10.18632/aging.100208

**Published:** 2010-10-14

**Authors:** Mohammad M. Rahman, Olga Stuchlick, Enas G. El-Karim, Ryan Stuart, Edward T. Kipreos, Lance Wells

**Affiliations:** ^1^ Department of Genetics, University of Georgia, Athens, GA 30602, USA; ^2^ Department of Biochemistry & Molecular Biology, Complex Carbohydrate Research Center, University of Georgia, Athens, GA 30602, USA; ^3^ Department of Cellular Biology, University of Georgia, Athens, GA 30602, USA

**Keywords:** C. elegans, lifespan, O-GlcNAc, OGT, OGA

## Abstract

O-linked-β-N-acetylglucosamine (O-GlcNAc) modification is a regulatory, nuclear and cytoplasmic post-translational glycosylation of proteins associated with age-related diseases such as Alzheimer's, Parkinson's, and type II diabetes. Global elevation of O-GlcNAc levels on intracellular proteins can induce insulin resistance, the hallmark of type II diabetes, in mammalian systems. In *C. elegans*, attenuation of the insulin-like signal transduction pathway increases adult lifespan of the nematode. We demonstrate that the O-GlcNAc cycling enzymes OGT and OGA, which add and remove O-GlcNAc respectively, modulate lifespan in *C. elegans*. Median adult lifespan is increased in an *oga-1* deletion strain while median adult life span is decreased upon *ogt-1* deletion. The O-GlcNAc-mediated effect on nematode lifespan is dependent on the FoxO transcription factor DAF-16. DAF-16 is a key factor in the insulin-like signal transduction pathway to regulate reproductive development, lifespan, stress tolerance, and dauer formation in *C. elegans*. Our data indicates that O-GlcNAc cycling selectively influences only a subset of DAF-16 mediated phenotypes, including lifespan and oxidative stress resistance. We performed an affinity purification of O-GlcNAc-modified proteins and observed that a high percentage of these proteins are regulated by insulin signaling and/or impact insulin pathway functional outcomes, suggesting that the O-GlcNAc modification may control downstream effectors to modulate insulin pathway mediated cellular processes.

## INTRODUCTION

Insulin resistance precedes and is a hallmark of type II diabetes [1]. Despite decades of progress in under-standing insulin-mediated signal transduction, the molecular mechanisms underlying insulin resistance are complex, and are not fully explored. The onset of insulin resistance is correlated with increased flux through the hexosamine biosynthetic pathway that leads to elevated UDP-N-acetylglucosamine (GlcNAc) from a glycolysis intermediate in mammalian cells [2]. UDP-GlcNAc is the donor for the post-translational modification of nuclear and cytosolic proteins through the addition of an O-linked GlcNAc molecule to serine and threonine residues [3]. Metazoa have conserved O-GlcNAc transferase (OGT) and O-GlcNAcase (OGA) enzymes that catalyze the addition and removal of O-GlcNAc, respectively. O-GlcNAc cycling occurs in both the nucleus and cytoplasm, and numerous proteins have been reported to be O-GlcNAc modified including transcription factors, metabolic enzymes, and kinases (reviewed in [4]). Elevation of O-GlcNAc levels, either pharmacologically or genetically, has been demonstrated in mammalian systems to induce insulin resistance. Aberrant O-GlcNAc modification is implicated in human diabetes, and age related neurodegenerative diseases [5-7].

**Figure 6.**
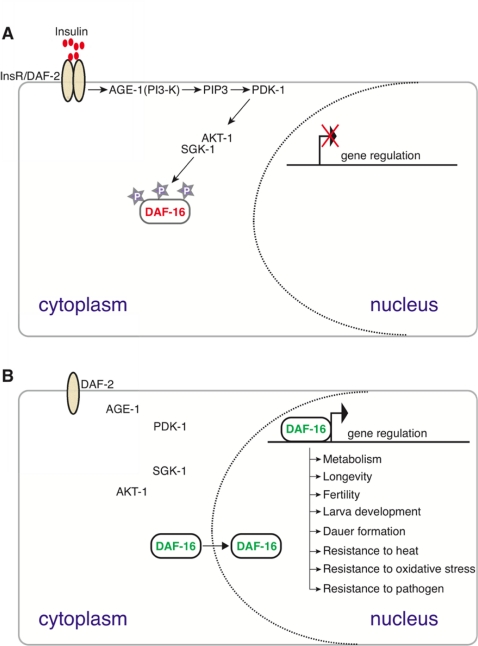
A conserved insulin signaling pathway in *C. elegans* regulates numerous functions including stress response, metabolism, dauer formation, and reproductive development by restricting the nuclear localization of the DAF-16/FoxO transcription factor upon nutrient availability [45]. Upon ligand binding (top panel), the insulin-like receptor DAF-2 activates the AGE-1 PI3 kinase that facilitates the activation of PDK-1 and AKT-1. AKT-1-mediated phosphorylation sequesters DAF-16 in the cytoplasm. In the absence of PI3K/AKT signaling (bottom panel) DAF-16 enters the nucleus and regulates the expression of target genes to mediate numerous DAF-16 dependent processes, including those listed.

In *C. elegans*, the conserved insulin-like signaling pathway regulates lifespan as well as reproductive development, stress response, and dauer formation [8,9]. Upon insulin binding, the insulin receptor (DAF-2) catalyzes receptor tyrosine phosphorylation events to activate phosphatidylinositol-3 kinase (PI3K/AGE-1) [10]. AGE-1 activates the effector kinases, AKT-1 and SGK-1, which antagonize the forkhead box O (FoxO) transcription factor DAF-16 by preventing its nuclear localization (see Figure 6). A reduction in DAF-2-mediated signaling allows DAF-16 to localize to the nucleus where it regulates a large number of genes essential for metabolism, energy storage, reproductive development, immunity, stress resistance, and other novel processes which collectively modulate lifespan in *C. elegans* [11-13]. Adult lifespan extension upon reduction in insulin signaling has also been reported in *Drosophila*, mice, and recently in a centenarian human population [14,15]. DAF-16 function is controlled by additional mechanisms beyond its nuclear localization, as neither constitutive nuclear localization nor over-expression is sufficient to induce maximum lifespan extension [16]. The mechanism by which insulin signaling mediated through FoxO/DAF-16 produces distinct biological outcomes in response to varying levels of insulin signaling remains ambiguous [12,17].

The *C. elegans* genome contains a single OGT ortholog, *ogt-1*, and a single OGA ortholog, *oga-1*. In the current study we demonstrate that the O-GlcNAc cycling enzymes modulate median adult lifespan in *C. elegans*. Intriguingly, we delineate downstream functions of the insulin signaling pathway that are O-GlcNAc dependent, and others that appear to be O-GlcNAc independent. Finally we used a biochemical approach to identify putative O-GlcNAc target proteins, and found that the majority of these proteins are regulated by insulin signaling, and a significant percentage functionally impact lifespan. Our work therefore suggests a potential mechanism by which the post-translational O-GlcNAc modification of proteins downstream of the insulin pathway could differentially modulate insulin-mediated signaling outcomes.

## RESULTS

### Cellular O-GlcNAc levels modulate adult lifespan in *C. elegans*

**Figure 1.**
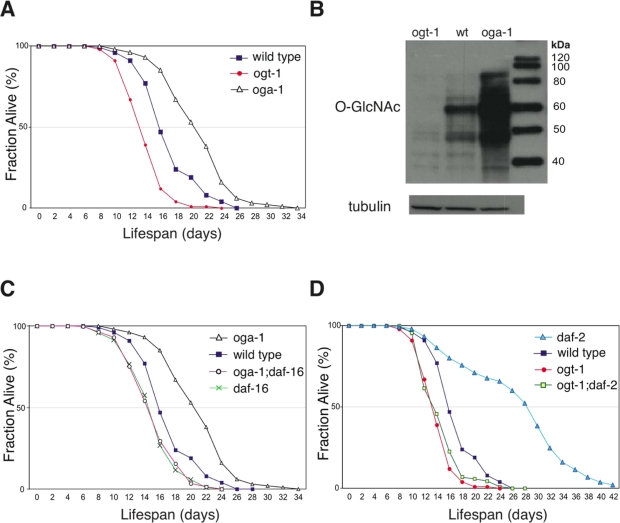
Elevated O-GlcNAc levels extend adult lifespan in *C. elegans.* (**A**) The *oga-1(ok1207)* mutant has an extended adult lifespan while the *ogt-1(ok1474)* mutant has a reduced lifespan relative to wild-type animals, assayed at 20°C. Lifespan curves are based on pooled data from multiple replicates described in Table 1. (**B**) OGA-1 and OGT-1 regulate cellular O-GlcNAc-modified protein levels. Western blot of total animal extract from *ogt-1(ok1474)*, wild-type, and *oga-1(ok1207)* animals probed with antibodies against O-GlcNAc and tubulin showing marked differences in total O-GlcNAcylated protein levels. (**C**) Lifespan extension in the *oga-1* mutant is DAF-16-dependent, as seen in the reduced lifespan of the *oga-1(ok1207); daf-16(mu86)* double mutant relative to the *oga-1(ok1207)* single mutant. (**D**) Loss of O-GlcNAc modification, in the *daf-2(e1370); ogt-1(ok1474)* double mutant, reduces the extended lifespan of the *daf-2(e1370)* mutant. Control curves are re-plotted to facilitate comparisons in panels **A**, **C**, and **D**.

In *C. elegans*, O-GlcNAcase (*oga-1*) and O-GlcNAc transferase (*ogt-1*) homozygous null mutants are viable and appear overtly wild type [18,19]. However, we observed that *oga-1(ok1207)* null mutant adults live ~33% longer than the wild-type animals, while *ogt-1(ok1474)* null mutants have a lifespan ~20% shorter than the wild type at 20°C (Figure 1A). In *C. elegans*, movement ability is used as a general index of vitality [20]. The *ogt-1* mutant animals appear to age prematurely, as exemplified by markedly slower movement at day 12 of adulthood relative to still-active wild-type and *oga-1* mutant adults (Suppl. Movie 1). *ogt-1* null mutant animals have normal developmental timing (43 ± 3.9 hrs vs. 41 ± 0.7 hrs for *ogt-1* and wild type, respectively, p = not significant, >0.5), and generate fertile progeny numbers similar to wild-type animals (263 ± 80 vs. 289 ±32 for *ogt-1* and wild type, respectively, p = ns), suggesting that the reduced movement and earlier death of *ogt-1* mutant adults is not due to a general ‘sickness’. The *oga-1(ok1207)* mutant demonstrates a substantial increase in O-GlcNAc-modified protein levels as determined by immunoblot of whole animal lysate, while the *ogt-1(ok1474)* mutant has only residual staining (Figure 1B). To determine whether adult lifespan extension in *oga-1* mutants arises from excessive O-GlcNAc-modifications, we analyzed the lifespan of the *oga-1*; *ogt-1* double mutant which has a markedly reduced level of O-GlcNAc-modified proteins similar to the *ogt-1* mutant (data not shown). The adult lifespan of the*oga-1; ogt-1* double mutant is similar to that of wild type (Table 1), confirming that lifespan extension observed in *oga-1* mutants is dependent on excessive O-GlcNAc-modified protein levels.

### O-GlcNAc cycling enzymes modulate insulin-mediated signaling in *C. elegans*

**Figure 2.**
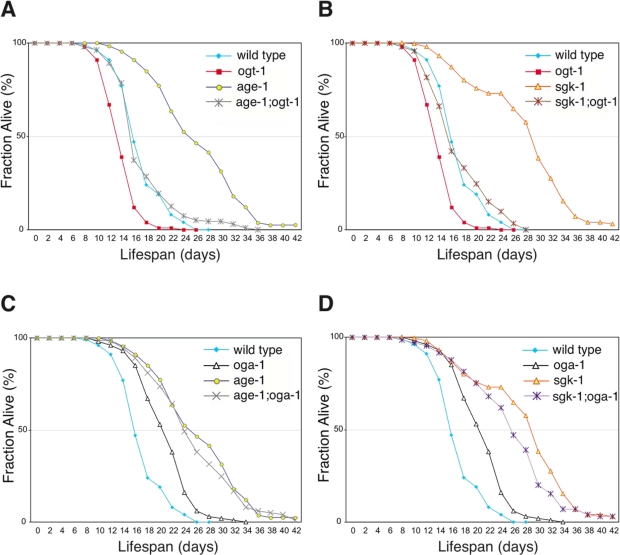
Elevated O-GlcNAc modification of proteins does not increase adult lifespan further in long-lived insulin signaling pathway mutant animals. (**A** and **B**) Lifespan of *age-1(hx546)* and *sgk-1(ok538)* mutants is dependent on protein O-GlcNAc modification as seen in *age-1; ogt-1* (**A**) and *sgk-1; ogt-1* (**B**) double mutants. (**C** and **D**) The adult lifespan extension associated with the *oga-1(ok1207)* mutant is not synergistic or additive with mutations of the long-lived insulin signaling pathway kinase *age-1* (**C**) or sgk-1 (**D**). Lifespan curves (**A-D**) are based on pooled data from multiple replicates as described in Table 1. Control curves are re-plotted to facilitate comparisons in panels **A-D**.

Inactivation of insulin-mediated signaling increases *C. elegans* adult lifespan in a DAF-16-dependent manner [21]. We observed that the lifespan extension in the *oga-1(ok1207)* mutant is also dependent on intact DAF-16 (Figure 1C). Inactivation of OGT-1 function in *daf-2(e1370)* mutants dramatically reduces its adult lifespan arguing that O-GlcNAc modification of cellular proteins is a requirement for the *daf-2* mutant long lifespan (Figure 1D). Lifespan extension regulated by insulin-mediated signaling in *C. elegans* is mainly achieved through its downstream effector kinases, such as AGE-1 and SGK-1 (Figure 6). Inactivation of the effector kinases AGE-1 and SGK-1 also results in significant adult lifespan extension [22,23]. To further test whether O-GlcNAc modification is essential for insulin signaling mediated adult lifespan regulation, we inactivated O-GlcNAc transferase function in both the long-lived *age-1(hx546)* and *sgk-1(ok538)* mutants. We observed that adult lifespan extension in *age-1* and *sgk-1* mutants are completely suppressed to wild-type levels upon combination with the short-lived *ogt-1(ok1474)* mutant allele (Figure 2A and 2B).

In a conventional genetic analysis, genes that function in parallel pathways with the same functional output generally demonstrate additive interactions. However, combining the long-lived *oga-1(ok1207)* mutant allele with long-lived insulin signaling pathway mutants, *daf-2*, *age-1*, or *sgk-1*, did not extend lifespan further, suggesting that O-GlcNAc-modified proteins function in the same genetic pathway (Figure 2C and 2D, Table 1). To further investigate where O-GlcNAc cycling impinges on the insulin signaling pathway, we analyzed both *akt-1* and *pdk-1* gain-of-function (gf) mutant alleles that constitutively activate the insulin signaling pathway [24,25]. Both *akt-1(mg144)gf* and *pdk-1(mg142)gf* mutant lifespans are shorter than that of wild type (Table 1). The combination of *akt-1(mg144)gf* or *pdk-1(mg142)gf* mutant alleles with the long-lived *oga-1(ok1207*) allele failed to suppress the lifespan extension associated with the *oga-1* mutant (Table 1). This data suggests that O-GlcNAc-modified proteins act downstream of the insulin pathway effector kinases to regulate insulin signaling outcomes. However both *mg144* and *mg142* alleles also do not suppress the extended lifespan of the *age-1(hx546)* mutant [24]; and thus this conclusion is not definitive.

**Figure 3.**
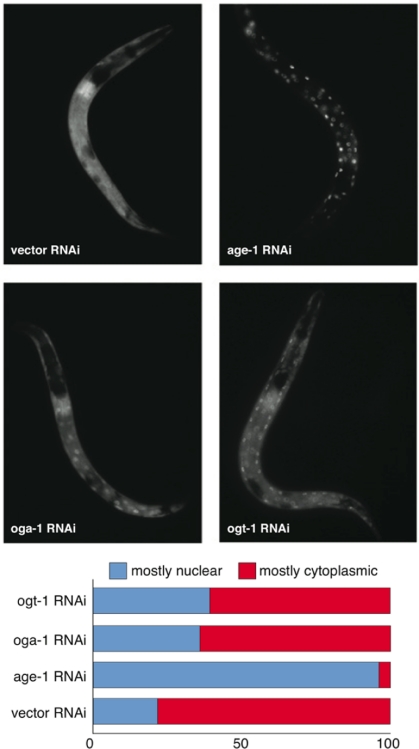
Aberrant protein O-GlcNAc cycling modestly enhances DAF-16 nuclear localization. DAF-16::GFP (translational fusion) nuclear localization in *oga-1(RNAi)* and *ogt-1(RNAi)* animals is modestly increased relative to control (vector) RNAi animals but much less than observed in *age-1(RNAi)* animals (upper panel). Lower panel is the quantification of DAF-16::GFP levels in intestinal cells upon RNAi inactivation of *ogt-1*, *oga-1*, *age-1*, and vector control.

In *C. elegans*, the insulin signaling pathway regulates numerous biological functions through DAF-16 by restricting its nuclear localization including, dauer entry, longevity, stress resistance, fertility, and reproductive development [12]. We sought to determine whether perturbing the O-GlcNAc cycling enzymes OGT-1 or OGA-1 would affect DAF-16 subcellular localization. Inactivation of the insulin signaling pathway effector kinase AGE-1 (by RNAi) produced virtually complete nuclear localization of DAF-16 (Figure 3). Interestingly, inactivation of either OGA-1 or OGT-1 produced a similar but modest increase in DAF-16 nuclear localization (Figure 3). The observation that inactivating either OGA-1 or OGT-1 produces similar effects on DAF-16 nuclear localization but opposite effects on insulin pathway outcomes, suggests that O-GlcNAc cycling does not functionally modulate the insulin pathway at the level of DAF-16 nuclear localization.

### Perturbation in O-GlcNAc cycling affects only a subset of insulin-mediated functions

**Figure 4.**
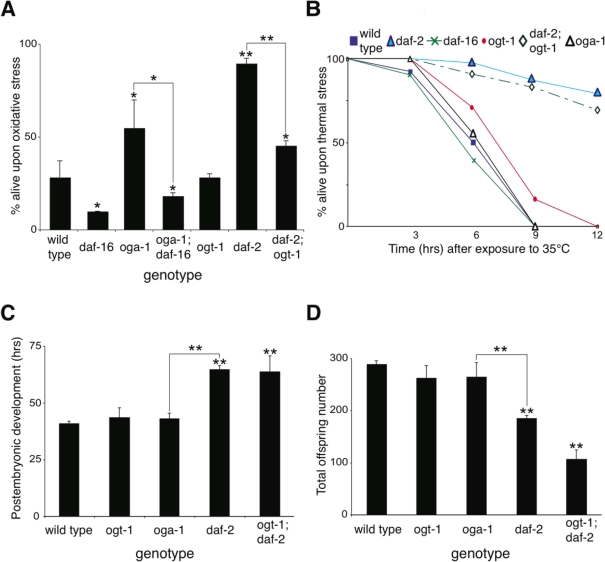
Inactivation of O-GlcNAc cycling enzymes only affects a subset of functions regulated by the insulin-like signaling pathway. (**A**) The *oga-1(ok1207)* mutant is resistant to oxidative stress (100mM paraquat for 9 hrs). Notably, the oxidative stress resistance in *daf-2(e1370)* mutant is significantly reduced in the *daf-2(e1370); ogt-1(ok1474)* double mutant, suggesting that protein O-GlcNAc modification contributes to the oxidative resistance of the *daf-2* mutant. (**B**) The long-lived *oga-1(ok1207)* mutant is sensitive to heat stress (35°C), unlike the long-lived *daf-2 (e1370)* mutant. (**C**) *oga-1* and *ogt-1* mutants have normal developmental timing [from hatch to the fourth larval molt] at 20°C. The *daf-2* mutant has delayed post-embryonic development, and this delay is unaffected by loss of O-GlcNAc modification in the *daf-2*; *ogt-1* double mutant. (**D**) *oga-1* and *ogt-1* mutants generate normal numbers of offspring while the *daf-2* mutant has reduced fecundity at 20°C. The reduced fecundity in the *daf-2* mutant is not rescued by loss of O-GlcNAc modification in the *daf-2*; *ogt-1* double mutant. Asterisks above bars denote statistically significant differences from wild type (* p < 0.05 and ** p < 0.01); while asterisks above solid lines connecting two genotypes denote statistically significant differences between those genotypes.

**Figure 5.**
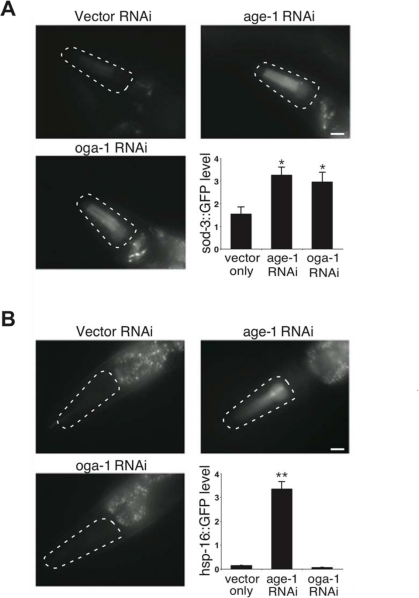
Aberrant protein O-GlcNAc cycling differentially affects distinct stress response pathways in *C. elegans*. (**A**) Images and quantification of *sod-3*::GFP reporter expression upon inactivation of OGA-1 and the insulin pathway effector kinase AGE-1. (**B**) Images and quantification of *hsp-16-2*::GFP reporter expression upon inactivation of OGA-1 and AGE-1. Note that inactivation of AGE-1 by RNAi induces both SOD-3 and HSP-16 expression, while OGA-1 inactivation only induces SOD-3 expression. Asterisks denote statistically significant differences from vector RNAi controls (* p < 0.05 and ** p < 0.01).

Lifespan extension in insulin pathway mutants is correlated with oxidative and thermal stress resistance as well as changes in animal development [26,27]. We observed that the long-lived *oga-1* mutant is resistant to oxidative stress in a DAF-16-dependent manner similar to that observed with the *daf-2* mutant (Figure 4A). Co-inactivation of OGT-1 and DAF-2 in *daf-2(e1370)*;*ogt-1(ok1474)* double mutant animals significantly reduced the oxidative stress resistance associated with the *daf-2* mutant (Figure 4A). This data implies that protein O-GlcNAc modification contributes to the oxidative stress tolerance that is prominent in insulin pathway mutants. In contrast to oxidative stress resistance, the long-lived *oga-1* mutant differs from long-lived *daf-2* mutants in that the *oga-1* mutant does not exhibit increased thermal stress tolerance, delayed developmental timing defects, or reduced fecundity, which are prominent in *daf-2* mutants (Figure 4B, 4C, and 4D, respectively) [16,26,28,29]. Oxidative stress resistance in the insulin pathway mutants arises from the DAF-16-mediated expression of several genes including superoxide dismutase SOD-3, while the increased thermotolerance is correlated with the induction of several heat-shock proteins, such as HSP-16 [27,30,31]. We observed that the inactivation of OGA-1 increased the expression of a *sod-3::GFP* reporter but not a *hsp-16.2::GFP* reporter (Figure 5A and 5B, respectively). Our data indicates that O-GlcNAc modification of proteins is critical for only a subset of the insulin pathway functional outcomes that are downstream of DAF-16.

**Table 1.** Adult Lifespan Analysis

Genotype	Median LS ±STDEV	*p*-value vs. wild type		Sample size n(censored)	Replicates N
		Log-rank	Wilcoxon		
wild type	15.9±3.6	-	-	404(59)	8
*oga-1(ok1207)*	20.9±4.1	<0.0001	<0.0001	317(43)	6
*ogt-1(ok1474)*	12.9±1.9	<0.0001	<0.0001	306(64)	6
*oga-1(ok1207); ogt-1(ok1474)*	15.5±4.9	0.030	0.040	174(35)	2
*daf-2(e1370)*	28.6±3.5	<0.0001	<0.0001	235(26)	3
*oga-1(ok1207); daf-2(e1370)*	21.6±0.6	<0.0001	<0.0001	201(20)	3
*ogt-1(ok1474); daf-2(e1370)*	13.2±1.9	<0.0001	<0.0001	218(28)	3
*daf-16(mu86)*	14.3±2.5	0.0001	<0.0001	228(60)	3
*oga-1(ok1207); daf-16(mu86)*	14.8±1.8	0.019	0.009	173(25)	2
*ogt-1(ok1474); daf-16(mu86)*	12.8±0.4	<0.0001	<0.0001	150(24)	2
*age-1(hx546)*	25.6±4.1	<0.0001	<0.0001	216(18)	3
*oga-1(ok1207); age-1(hx546)*	24.7±6.8	<0.0001	<0.0001	241(17)	3
*ogt-1(ok1474); age-1(hx546)*	15.9±1.6	0.424	0.590	258(31)	3
*sgk-1(ok538)*	29.8±3.2	<0.0001	<0.0001	235(16)	3
*oga-1(ok1207); sgk-1(ok538)*	26.2±2.1	<0.0001	<0.0001	209(30)	3
*ogt-1(ok1474); sgk-1(ok538)*	16.6±2.3	0.343	0.375	214(39)	3
*pdk-1(mg142)gf*	14.8±1.7	0.013	0.007	334(84)	5
*oga-1(ok1207); pdk-1(mg142)*	18.7±8.1^a^	<0.0001	<0.0001	284(43)	5
*akt-1(mg144)gf*	11.5±2.1	<0.0001	<0.0001	205(32)	2
*oga-1(ok1207); akt-1(mg144)*	19.8±4.2^b^	0.0002	0.0002	229(22)	2

a. *pdk-1* vs. *oga-1;pdk-1* p-value <0.0001

b. *akt-1* vs. *oga-1;akt-1* p-value <0.0001

N = number of replicates; n = pooled animals from N replicate experiments (censored animals were included in the Log-rank/Wilcoxon analysis)

### Identification of putative O-GlcNAc target proteins through affinity purification

In an effort to identify the proteins that are modified by O-GlcNAc in *C. elegans*, we affinity purified O-GlcNAc-modified proteins from *oga-1(ok1207)* and *ogt-1(ok1474)* mutant adults, and then identified the affinity purified proteins by liquid chromatography coupled to tandem mass spectrometry (LC-MS/MS). Non-specific proteins would be expected to be present in both purifications, while O-GlcNAc-modified proteins (or proteins tightly-associated with O-GlcNAc-modified proteins) would be specific for the *oga-1* mutant lysate. Proteins that were identified from affinity purifications of both *oga-1* and *ogt-1* mutant lysates were censored as non-specific. Using this approach, we identified 13 distinct proteins that were specific for the O-GlcNAc affinity purification from *oga-1* mutant lysate (Table 2). The 13 proteins encompass a total of 21 proteins when including paralogs with identical or nearly identical protein sequences that could not be distinguished by mass spectrometry. The majority of the putative O-GlcNAc target proteins are regulated by the insulin signaling pathway and a substantial percentage functionally modulate lifespan or oxidative stress (Table 2 and discussion below), suggesting that OGT-1 and OGA-1 may control a subset of insulin signaling functional outcomes by directly targeting the O-GlcNAc modification of proteins that act downstream of the insulin signal transduction pathway.

## DISCUSSION

In *C. elegans*, O-GlcNAc cycling enzyme mutants are viable and therefore provide a useful model to elucidate the role of O-GlcNAc protein modifications, which have been implicated in numerous cellular processes. In our current study we demonstrated that a loss of O-GlcNAc transferase (OGT-1) activity reduces adult lifespan in the nematode *C. elegans*. Conversely, inactivation of O-GlcNAcase (OGA-1) leads to a moderate increase in adult lifespan. While the accumulation of excessive levels of O-GlcNAc-modified proteins in *oga-1* mutant animals is associated with lifespan extension, it is not associated with obvious abnormalities in reproductive development, as *oga-1* mutants have an overtly wild-type appearance, develop normally into adults, and generate normal numbers of fertile offspring. Similarly, lifespan reduction in *ogt-1* null mutants does not appear to be due to a general ‘sickness’ of the mutant animals, as *ogt-1* mutants also develop normally into fertile adults, and generate normal numbers of offspring (Figure 4).

Love et al. recently reported similar results that OGT-1 inactivation shortens both wild-type and *daf-2* mutant lifespans [32]. However, their findings differ from ours in that they reported that OGA-1 inactivation did not significantly affect lifespan in a wild-type background but slightly extended lifespan in a *daf-2* mutant background [32]. We observed that *oga-1* mutant lifespan was significantly longer than that of wild type (three independent experiments showed significant differences; p<0.0001, n=66-89 for each experiment); and double mutant analysis of *oga-1* with each of three separate components of the insulin signal transduction pathway, *daf-2*, *age-1*, and *sgk-1*, did not show additional lifespan increases in our experiments.

The failure to observe additive lifespan extension when combining *oga-1* and insulin pathway mutations is consistent with O-GlcNAc-modified proteins working in the same genetic pathway as insulin signaling. In *C. elegans*, insulin signaling regulates gene expression by restricting DAF-16, whose activity is essential for the extended lifespan that is observed in insulin pathway mutants [11,12]. We found that DAF-16 is required for the lifespan extension in *oga-1* mutant animals, which is also consistent with O-GlcNAc cycling functioning in the same genetic pathway as insulin signaling.

The observation that elevated O-GlcNAc levels (in the *oga-1* mutant) mimics only a subset of insulin pathway mutant phenotypes (lifespan extension and resistance to oxidative stress) but not others (delayed developmental timing, reduced fecundity, and increased thermotolerance) suggests that O-GlcNAc cycling only alters a subset of functional outcomes under the control of insulin-mediated signaling. The insulin signaling pathway regulates DAF-16 nuclear localization. If OGA-1 and OGT-1 impacted the insulin pathway upstream of DAF-16, it would be expected that *oga-1* and *ogt-1* mutants would have opposite effects on DAF-16 nuclear localization. However, we observed that both mutants have the same modest enhancement of DAF-16 nuclear localization, suggesting that O-GlcNAc cycling modulates insulin signaling outcomes downstream of DAF-16.

We performed an affinity purification of O-GlcNAc-modified proteins that identified 13 proteins (Table 2). The mammalian counterparts of 6 of the 13 proteins are known to be O-GlcNAc modified, suggesting that these are conserved targets of O-GlcNAc transferase (Table 2).

Strikingly, the expression of 10 of the 13 putative O-GlcNAc target proteins is controlled by the insulin signaling pathway (Table 2). More significantly, 4 of the 13 putative O-GlcNAc target proteins have been reported to modulate lifespan in *C. elegans*. Inactivation of either of the F0F1-type ATP synthases ATP-2 or H28O16.1 is observed to increase adult lifespan and increase the expression of a *sod-3*::GFP reporter [33]. Inactivation of the glyceraldehyde-3-phosphate dehydrogenase GPD-2 further extends the lifespan of *daf-2* mutants [34]; while inactivation of the malate dehydrogenase MDH-1 shortens *daf-2* mutant lifespan [35]. Additionally, inactivation of GPD-2 and GPD-3 allows the *daf-2(e1370)* mutant to escape dauer entry, thereby demonstrating a critical role for GPDs in regulating one of the functional outcomes under the control of the insulin signaling pathway [36]. Our work therefore demonstrates that downstream targets of the insulin pathway are O-GlcNAc modified. This provides the possibility that O-GlcNAc cycling enzymes act downstream of DAF-16 to directly functionally modulate specific insulin-regulated processes.

In mammals, increasing O-GlcNAc levels by reducing O-GlcNAcase (OGA) activity or increasing O-GlcNAc transferase (OGT) activity leads to insulin resistance [37]. Increased O-GlcNAc levels inhibit insulin stimulated activation of AKT, and disrupt insulin-mediated glucose transport in adipocytes demonstrating a direct involvement of O-GlcNAc cycling in the attenua- tion of insulin-mediated signaling functional outcomes. A reduced ability of insulin to activate glucose transport into cells, i.e., insulin resistance, is marked by high glucose and high insulin levels in circulating blood, and aberrant insulin-regulated gene functions [10]. Abnormalities in carbohydrate and fat metabolism are characteristic of insulin-resistant tissues, and are implicated in elevated plasma sugar and fatty acid levels in patients with type II diabetes [38]. In *C. elegans*, similar disarray in carbohydrate and fat storage is observed in insulin pathway mutant animals [9]. Previous studies also demonstrated abnormal carbohydrate and fat storage upon disruption of O-GlcNAc cycling [18]. Therefore, O-GlcNAc deregulation in *C. elegans* is strikingly similar to the insulin-resistant phenotype previously described in mammalian studies despite the absence of a blood circulation system and the consequent need for blood glucose homeostasis in the nematode.

**Table 2.** Summary of putative *C. elegans* O-GlcNAc targets

Proteins	Molecular Identity	Expression in *daf-2* mutant	O-GlcNAc modified in vertebrate homologs
PHI-37	F0F1-type ATP synthase	Downregulated [46]	NR
ATP-2	F0F1-type ATP synthase	Downregulated [46]	NR
MDH-1	Malate dehydrogenase	Downregulated [47]	MDH1 [7]
GPD-2, GPD-3	Glyceraldehyde-3-phosphate dehydrogenase, major isozyme	Upregulated [48,49]	GAPDH [7,50]
GPD-1, GPD-4	Glyceraldehyde-3-phosphate dehydrogenase, minor isozyme	Downregulated [46]	GAPDH [7,50]
EFT-3, EFT-4	Translation elongation factor 1 alpha	Downregulated [46]	EEF1AO [50]
ACT-1, ACT-2, ACT-3, ACT-4	Actin	Downregulated [46]	ACTG1 [7]
TBA-1, TBA-2, TBA-4	Alpha tubulin	Downregulated [46]	TUBA1A [7]
VIT-6	Vitellogenin	Downregulated [46]	NR
VHA-13	Vacuolar protein translocating ATPase	NR	NR
CDC-48.2	AAA-type ATPase	NR	NR
F27D4.1	Electron transfer flavoprotein	NR	NR
C16A3.10	Ornithine aminotransferase	Downregulated [48]	NR

EFT-3 and EFT-4 have identical protein sequences, as do GPD-2 and GPD-3. The peptides identified by LC-MS/MS did not distinguish between the almost identical ACT-1, ACT-2, ACT-3, and ACT-4; TBA-1, TBA-2, and TBA-4; or GPD-2 and GPD-3, respectively. NR, not reported.

Future studies will be required to establish the effects that O-GlcNAc modifications have on the target proteins that were identified in this study, and the functional significance of the modifications on specific insulin pathway outcomes. Many of the O-GlcNAc targets appear to be conserved between nematodes and vertebrates, and it will be interesting in the future to determine whether O-GlcNAc cycling acts downstream of the insulin pathway in vertebrates to control subsets of FoxO-dependent processes. The use of *C. elegans* as an amenable genetic model will allow a fuller exploration of the complexities of O-GlcNAc modification and FoxO-mediated gene expression, with implications for human neuro-degenerative diseases, diabetes, and developmental processes including aging.

## METHODS

### Nematode strains

The following nematode strains were used in this study: N2 wild type Bristol, RB1342 *ogt-1(ok1474)*, RB1169 *oga-1(ok1207)*, CF1041 *daf-2(e1370)*, CF1380 *daf-16(mu86)*, TJ1052 *age-1(hx546)*, VC345 *sgk-1(ok538)*, GR1310 *akt-1(mg144)*, GR1318 *pdk-1(mg142)*, and ET422 *muIs109[Pdaf-16::*GFP::DAF-16 cDNA + *Podr-1*::RFP], CF1553 *muIs84*[pAD76 *(sod-3::GFP)*], CL2070 *dvls70*[integrat-ed*hsp-16-2::*GFP+pRF4], ET434 *oga-1(ok1207)*, ET435 *ogt-1(ok1474),* ET436 *oga-1(ok1207)*; *ogt-1(ok1474)*, ET439 *oga-1(ok1207)*; *daf-2(e1370)*, ET438 *ogt-1(ok1474)*; *daf-2(e1370)*, ET437 *oga-1(ok1207)*; *daf-16(mu86)*, ET468 *ogt-1(ok1474)*; *daf-16(mu86)*, ET469 *oga-1(ok1207)*; *age-1(hx546)*, ET393 *ogt-1(ok1474)*; *age-1(hx546)*, ET471 *oga-1(ok1207)*; *sgk-1(ok538)*, ET470 *ogt-1(ok1474)*; *sgk-1(ok538)*, ET472 *oga-1(ok1207)*; *pdk-1(mg142)*, and ET445 *oga-1(ok1207)*; *akt-1(mg144)*. The *oga-1(ok1207)* and *ogt-1(ok1474)* mutants in strains ET434 and ET435 were outcrossed 6 times against N2 in our laboratory with the mutations followed by PCR. The mutation in ogt-*1(ok1474)* and *oga-1(ok1207)* alleles were verified using PCR primers provided by the *C. elegans* Gene Knockout Project at OMRF, which is part of the International *C. elegans* Gene Knockout Consortium. The mutations in double mutant strains were followed by PCR and restriction enzyme analysis or by DNA sequencing spanning the region corresponding to the mutations, where appropriate.

### Lifespan analysis

All lifespan assays were performed using 85-125 animals for each genotype per assay at 20°C on NGM plates seeded with OP50 bacteria following established procedures [8]. Adults were transferred to fresh plates every alternate day during the fertile period, and later when necessary. Adults were counted as dead when they failed to respond to repeated head and tail prodding. Multiple assays were pooled for final analysis.

### Statistical analyses

Survival curves were analyzed by the Log-rank (Mantel-Cox) and Wilcoxon tests using GraphPad Prism (version 5.0) to determine the significance between mutant and wild-type controls as indicated in Table 1. In other experiments, statistical significance was determined with the unpaired two-tailed Student's t-test.

### Stress tolerance assay

Oxidative stress resistance assays were performed in multi-well plates by immersing adults in M9 media containing 100 mM paraquat (N,N'-dimethyl-4,4'-bipyridinium dichloride) using 10-20 animals for each genotype per assay at 20°C [39,40]. A complete absence of swimming movement was scored as death, and also reconfirmed by transferred animals not moving upon prodding on agar plates. Death was scored every 3, 6, 9, and 12 hrs. Experimental data from multiple assays were pooled together for final analysis. Thermal resistance assays were performed on NGM plates pre-heated at 35°C using at least 50 animals for each genotype per assay [29,41]. Adults were incubated at 35°C, and were scored every hour by response to touch until all animals were dead. Experimental data from multiple assays were pooled together for final analysis.

### Developmental timing and fecundity assays

Postembryonic developmental timing was assayed by collecting comma-stage embryos from animals maintained at 20°C. The exact time of hatching was recorded, and then animals were transferred onto separate OP50-seeded NGM plates. After the third molt, animals were inspected more frequently (every 15 mins) to record the exact time of the fourth molt. The assay was repeated several times using at least 10 animals for each genotype per assay, and multiple experimental data were pooled together for final analysis. For fecundity analysis, individual larval stage animals were placed on OP50-seeded NGM plates at 20°C. Offspring were counted by moving them onto a fresh plate one-by-one. The assay was repeated multiple times with at least 10 animals per genotype in each assay, and data were pooled together for final analysis.

### Western immunoblotting

Adult animals were flash frozen in liquid nitrogen in lysis buffer (50mM HEPES pH 7.8 and 300mM NaCl). Frozen worms were crushed in a pestle pre-chilled with liquid nitrogen. The frozen ground worms were re-suspended in cold lysis buffer (with DNaseI and protease inhibitor cocktail from Roche) and subjected to sonication for 6-8 seconds followed by centrifugation at 16,000 rpm for 1 hour at 4°C. The middle portion (not including the pellet or floating lipids) of each tube was collected for analysis. An equal amount of whole animal lysate was separated on a 10% NuPAGE Bis-Tris gel (Invitrogen) followed by transfer onto a PVDF membrane (Millipore). The membrane was probed with anti-O-GlcNAc antibody from Affinity Bioreagents (1:2000 in 3% BSA dissolved in 1XTBS + 0.05% Tween20). The immunoblot was visualized using a chemiluminescent substrate (Super Signal West Pico from Pierce). The same membrane was re-probed with anti-α-tubulin antibody (Sigma) to normalize protein loading.

### RNAi experiments

All RNAi experiments were performed at 20°C unless otherwise mentioned using *E. coli* strain HT115 transformed with pPD129.36 clones from an RNAi library provided by Julie Ahringer for gene inactivation experiments, as previously described [42,43].

### GFP reporter assays

Experimental animals for analysis were grown on feeding RNAi for more than one generation. All animals were imaged at single shutter setting, and equal nuclear and cytoplasmic areas were chosen for light intensity measurement using OpenLab 5.5 software. The average of three mean light intensity measurement values per cell (in arbitrary units) for both nuclear and cytoplasmic regions were recorded for multiple cells in each treated and untreated animals. The assay was repeated several times with at least 10 animals per genotype in each assay at 20°C. Data from multiple experiments were pooled for final analysis. Both*sod-3::gfp* and *hsp-16-2::gfp* reporters were assayed in 10-day-old adults.

### Affinity purification and identification of O-GlcNAc-modified proteins

Adult animals were collected and flash frozen in liquid nitrogen in 50mM HEPES pH 7.8 and 50mM NaCl buffer. Frozen worms were crushed with a mortar and pestle pre-chilled with liquid nitrogen. The frozen ground worms were re-suspended in the above buffer (with DNaseI and protease inhibitor cocktail from Roche), and disrupted by sonication followed by centrifugation at 16,000 rpm for 1 hour at 4°C. Clear supernatant was collected and O-GlcNAc-modified proteins were identified as previously described [44]. Briefly, affinity enrichment with mAb14 to O-GlcNAc-modified proteins was conducted followed by the enriched proteins being reduced, alkylated, and trypsin digested. Protein assignments were made following tandem mass spectrometry on a linear ion trap using the *C. elegans* protein database from NCBI with filtering for a false-discovery rate of less than 1%, as previously described [44]. The number of peptides leading to the identification of each protein described in Table 2 are as follows: PHI-37 (8); MDH-1 (7); ATP-2 (3); GPD-1 (2); GPD-2 (4); ACT-1 (5); TBA-2 (3); EFT-3 (6); VIT-6 (2); VHA 13 (2); CDC-48.2 (2); F27D4.1 (2); and C16A3.10 (3).

## SUPPLEMENTAL MATERIAL

Supplemental Movie 1. Premature aging in animals lacking O-GlcNAc-modified proteins Three 10-second movie recordings, from left to right, *oga-1(ok1207)*, wild type (N2), and *ogt-1(ok1474)* animals at day 12 of adulthood. Note that, while *oga-1* mutant and wild-type animals are highly active, the *ogt-1* mutant animal is considerably slower, a phenotype similar to that of older (day 18) wild-type animals (data not shown). At an earlier time point (day 6) all three genotypes had indistinguishable movement (data not shown).

## Abbreviations

O-GlcNAc: O-linked b-N-acetylglucosamine
OGT: O-GlcNAc transferase
OGA: O-GlcNAcase (neutral b-Nacetylglucosaminidase)
PI3K: phosphatidylinositol-3-OH kinase
